# Fetal Head Growth during Early to Mid-Gestation Associated with Weight Gain in Mothers with Hyperemesis Gravidarum: A Retrospective Cohort Study

**DOI:** 10.3390/nu12061664

**Published:** 2020-06-03

**Authors:** Mitsue Muraoka, Koichiro Takagi, Mariko Ueno, Yoshihiro Morita, Hiroaki Nagano

**Affiliations:** 1 Department of Obstetrics and Gynecology, Shiseikai-daini Hospital, Tokyo 157-8550, Japan; muraoka.mitsue@twmu.ac.jp; 2 Department of Obstetrics and Gynecology, Tokyo Women’s Medical University, Medical Center East, Tokyo 116-8567, Japan; ueno.mariko@twmu.ac.jp (M.U.); morita.yoshihiro@twmu.ac.jp (Y.M.); nagano.hiroaki@twmu.ac.jp (H.N.)

**Keywords:** fetal growth, hyperemesis gravidarum, neurodevelopment

## Abstract

The epigenetic impact of malnutrition in mothers with hyperemesis gravidarum (HG) on their offspring has not been fully elucidated. Recently, several reports have demonstrated that children born to mothers with HG were small for gestational age and had low birth weight, reduced insulin sensitivity, and neurodevelopmental delays during childhood. Therefore, we examined the relationship between fetal growth and changes in the maternal body weight in HG cases. A total of 34 patients with HG were hospitalized and delivered at term between 2009 and 2012. The records of 69 cases of pregnant women without a history of HG were extracted after matching their maternal age, parity, pregestational body mass index (BMI), gestational age, and fetal sex ratio with those of the HG group for comparison. The maternal weight gain at term was less in the HG than in the control group. There was no statistical difference in birth weight, placental weight, and ultrasonic fetometric parameters expressed in standard deviation (SD) scores, including biparietal diameter, abdominal circumference, and femur length, between the HG and the control group. Whereas fetal head growth in the HG group was positively associated with maternal weight gain at 20 weeks of gestation only, this association was not observed in the control group. We herein demonstrate that maternal weight gain from the nadir is associated with fetal head growth at mid-gestation. Thus, maternal undernutrition in the first trimester of pregnancy could affect fetal brain growth and development, leading to an increased risk of neurodevelopmental delays in later life.

## 1. Introduction

Hyperemesis gravidarum (HG) involves persistent severe nausea and vomiting in pregnant women, affecting both the mother and the fetus [1]. The incidence of HG varies from country to country, ranging from 0.3% in Sweden to 1.2% in the United States and 3.6% in Japan [2,3,4]. The adverse effects caused by HG include dehydration, vitamin deficiency, and electrolyte imbalance in pregnant women [5] and an increased risk of low birth weight and small size for gestational age in the fetus [6]. In addition, HG may affect maternal acceptance of pregnancy as well as acceptance of motherhood and later quality of life [7]. With regard to the etiology of HG, a genetic study revealed a variance in the gene encoding an intracellular calcium release channel involved in vomiting and cyclic vomiting syndrome in families with possible inheritance of HG [8]. Moreover, through a genome-wide association study in humans, the placenta and appetite genes *GDF15* and *IGFBP7* were shown to be associated with HG [9,10]. Since the concept of the developmental origin of adult diseases has been introduced by accumulating epidemiological research, undernutrition during pregnancy, particularly during the first trimester, is known to be related to the future development of adult-onset disorders, such as obesity, cardiovascular disease, and diabetes in the early pregnancy [11]. Grooten et al. pointed out the relationship between early pregnancy, severe maternal weight loss, and elevation of blood pressure in the offspring as early as at 5–6 years of age [12]. Moreover, HG may result in reduced insulin sensitivity and neurodevelopmental delays at school age in offspring of mothers who experienced HG during pregnancy [13]. The adverse clinical effects of maternal undernutrition on neurodevelopmental growth, including those resulting from HG, have been reported. Fejzo et al. report that children born to mothers with HG are more likely to have neurodevelopmental problems, including attention disorders, learning delays, sensory disorders, and speech/language development delays [14]. Offspring born to mothers experiencing HG are also at an increased risk of behavioral and emotional disorders when they become adults [15].

To explore specific growth patterns of the fetus in pregnant mothers with HG, we investigated the association between fetal growth parameters and maternal body weight by analyzing ultrasonographic fetometric measurements.

## 2. Materials and Methods

Between 2009 and 2012, 34 women having HG with singleton pregnancies, who required hospitalization and delivered at full term (37 weeks 0 day to 41 weeks 6 days of gestation), were identified from 2778 recorded deliveries in the hospital records. This study received approval from the Institutional Review Board of Tokyo Women’s Medical University (REC no. 2208, June 2011). We diagnosed HG in the women if two of the following criteria were present: weight loss greater than 5% in the first trimester, ketone bodies in the urine, or the inability for food and fluid intake [16]. Prior to hospitalization, all patients were instructed by midwives and obstetricians to have frequent balanced meals, including low-fat and high-protein foods, and separate liquids and solids. On hospitalization, most patients were managed by peripheral intravenous administration of fluids, electrolytes, glucose, and vitamins in addition to oral food intake. The control group consisted of 69 cases who were matched by age, parity, pre-pregnancy body mass index (BMI), gestational age at delivery, and neonatal gender ratio. Maternal body weight was measured upon every perinatal visit without shoes and heavy clothing. The weight gain from the nadir was defined as the difference between the body weight at 20 weeks of gestation and the lowest body weight before admission. Ultrasonographic fetometry was performed on a GE Voluson S8 (GE Healthcare Japan, Tokyo). The clinical background characteristics of the participants in both groups are shown in Table 1. Fetal growth parameters including biparietal diameter (BPD), abdominal circumference (AC), femur length (FL), and estimated fetal body weight (EFW) were measured at approximately 20, 30, and 36 weeks of gestation and expressed as standard deviation (SD) scores. The mean values were subtracted from each measured value and divided by the given SD in each group. These values were compared statistically using JMP software, version 11, by Wilcoxon/Kruskal–Wallis and regression analyses. The statistical significance was set at *p* < 0.05.

## 3. Results

The clinical background characteristics of the participants in both groups are shown in Table 1. Ethnicity, smoking history, maternal age, parity, pre-gestational BMI, gestational age at delivery (weeks), birth weight, gender ratio, placental weight, and the weight ratio of the neonate to the placenta were not statistically significant. Maternal weight gain, measured at 20 weeks of gestation and at term, was significantly smaller in the HG group than in the control group. However, the birth weight was not statistically different between the two groups.

The clinical background of mothers with HG is shown in Table 2. Ultrasonographic fetometric data are shown in Table 3. BPD, AC, FL, and EFW of the HG group and control group were not significantly different at 20 weeks, 30 weeks, and 36 weeks of gestation.

**Figure 1:**
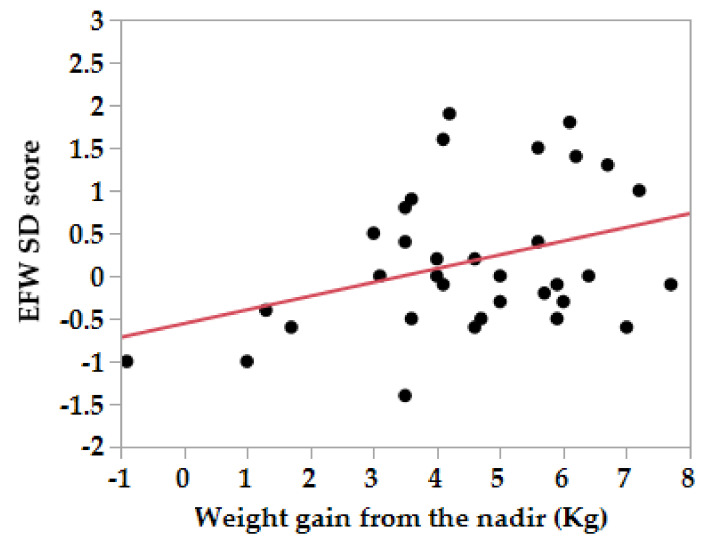
Correlation between SD scores of the estimated fetal body weight at 20 weeks of gestation in mothers with hyperemesis and maternal weight gain from the lowest body weight to the weight at 20 weeks of gestation. EFW SD score = −0.56 + 0.16 × Weight gain from the nadir (Kg). EFW, estimated fetal body weight.

**Figure 2:**
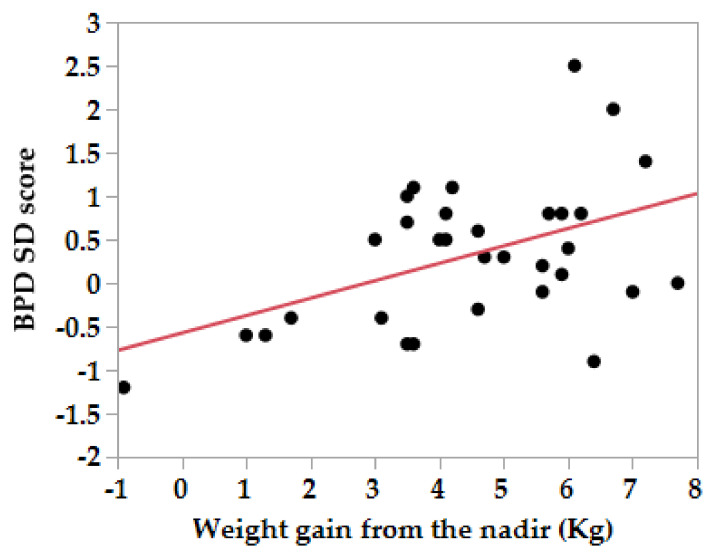
Correlation between SD scores of the biparietal diameter at 20 weeks of gestation in mothers with hyperemesis gravidarum and maternal weight gain from the lowest body weight to the weight at 20 weeks of gestation. BPD SD score = −0.57 + 0.20 × Weight gain from the nadir (Kg). BPD; biparietal diameter.

With regard to the association between maternal weight gain and fetal growth parameters in each trimester of gestation, the SD score of the BPD and EFW at 20 weeks of gestation were positively associated with the weight gain from the nadir (Figure 1) and the weight gain from the pre-pregnancy period (Figure 2) in the HG group, while the maternal weight gain was positively associated only with the AC at 30 to 36 weeks of gestation in the control group (Table 4).

## 4. Discussion

Main Findings

This retrospective study reveals that fetal head growth evaluated with ultrasound fetometry is positively correlated with maternal weight gain from both the pre-pregnancy period and the nadir in the first trimester of gestation in women with hyperemesis gravidarum. The same association was not observed in the matched control group.

### 4.1. Strengths and Limitations

The strengths of our study include the comparisons made with the matched control group. Although our sample was small, both the correlation coefficient and the statistical significance were high. As this study is based on data collected retrospectively during routine clinical services, we measured ultrasound fetometric data as SD scores to compare the outcomes of the two groups. The limitations of this study are the accuracy and reliability of the pre-pregnancy body weight values, as they were self-reported, and the lack of details of the nutritional support before and during hospitalization. However, our findings show that weight gain from the nadir and ultrasound fetometric fetal growth parameters are still highly associated.

### 4.2. Interpretation

Our study is the first to indicate an association between the growth of the fetal head and maternal body weight gain from pre-pregnancy and from the nadir in pregnant mothers with HG at mid-gestation. The same association between fetal head growth and the increase in maternal body weight was not observed in the matched control group. Why the effect of maternal undernutrition was only observed in the HG group has not been elucidated. However, it has been suggested that there may be a nutritional threshold that needs to be met to satisfy the nutritional demand for brain growth and development of the fetus. 

Only a few reports discussed the relationship between maternal nutrition and fetal brain growth in humans. Baker et al. reported that the head circumference of a neonate is positively associated with the ponderal index when maternal nutrition during pregnancy is adequate [17]. However, this association was not demonstrated in a group with a heavier placenta in which maternal nutrition was supposedly poor during pregnancy. Hinkle et al. reported similar findings to ours, demonstrating that fetal BPD and femur length were positively associated with maternal weight gain only at 17 weeks of gestation in women having a high risk of developing small for gestational age baby, including smoking during pregnancy, history of prior low birth weight delivery, low pre-pregnancy body weight, and complications of hypertension and chronic renal diseases [18]. It is understood that in situations where the feto–maternal nutritional transport is restricted, brain growth would be secured in the first instance. The effects of maternal nutritional restriction on the fetal brain have been shown previously in an animal model. Ma et al. report that in early to mid-gestational nutrient-restricted ewes, the brain weight of the fetus was transiently reduced at mid-gestation and returned to normal levels at term [19]. With regard to the qualitative effect of malnutrition on the brain, Edlow et al. showed that differences in the relative deficiency in micronutrients including minerals, vitamins, and amino acids provoked differential expression of more than 1000 genes in the mice embryonic brain, such as brain-derived neurotrophic factor (BDNF) and Kruppel-like factor 3 (KLF3), a transcription regulator linked to various neurological disorders [20]. In addition, protein restriction during the perinatal period is known to impair hippocampal development, leading to reduced BDNF levels. These animal studies suggested that the mechanism by which brain development is affected by under- or malnutrition is not related to caloric intake but to the intake of proteins and micronutrients including vitamins. 

The intake of micronutrients, vitamin B_12_, and folate in early pregnancy is demonstrated to be associated with cardiometabolic risk in the offspring at the age of 5–6 years in humans [21]. Although evidence of the effects of nutritional restriction on the human fetal brain is lacking, adverse clinical effects of maternal undernutrition on neurodevelopmental growth, including those resulting from HG, have been reported. Fejzo et al. reported that children born to mothers with HG are more likely to have neurodevelopmental problems, including attention disorders, learning delays, sensory disorders, and speech/language development delays [14]. Offspring born to mothers experiencing HG are also at an increased risk of behavioral and emotional disorders when they become adults [15]. Vitamin deficiency as an etiology of neurodevelopmental problems in HG remains to be elucidated, because in most HG cases, vitamin B group, vitamin C, and folate are routinely administered. If that is the case, we can speculate that another deficiency, such as amino acid deficiency, may be a candidate for the neurodevelopmental problems in HG cases.

Embryologically, the early to mid-gestation period is a critical period for brain growth, with the earliest synapses in the spinal cord developing during early gestation at 6 to 7 weeks [22]. Furthermore, subcortical structures have been shown to develop around mid-gestation at 12 to 22 weeks [23]. Nonetheless, the brain metabolism of the fetus has not been studied. Expanding on the existing body of evidence, our observation of a significant correlation between fetal head size and weight gain in mothers experiencing HG may be a sign of a morphological change in the fetal brain in response to maternal undernutrition. Our observation that fetal head growth was temporarily associated with maternal nutritional status may only be the coincidental sign of this morphological change in the fetal brain.

In a rat model, adverse effects of malnutrition on the fetus, other than brain development issues, include an increased risk of prostate disorders in later life [24]. Although such effects have not been demonstrated in humans, in cows, restricting nutrition shortly before conception until the end of the first trimester of pregnancy results in a decreased number of postnatal follicles, eventually leading to subfertility [25].

## 5. Conclusions

In this study, we demonstrated that maternal weight gain from the nadir was associated with fetal head growth at mid-gestation. Moreover, maternal undernutrition in the first trimester of pregnancy could affect fetal brain growth and development, leading to an increased risk of neurodevelopmental delays in later life. Further studies, including experiments using animal models, are needed to clarify the relationship between undernutrition in the first trimester of pregnancy and neurodevelopmental problems in later life. 

## Figures and Tables

**Table 1:** Clinical background of the HG and control groups.

Group	HG (*n* = 34)	Control (*n* = 69)	*P*-Value
Ethnicity	Japanese 33, Burmese 1	Japanese 69	0.716
Smoking before pregnancy	1	0	0.716
Smoking during pregnancy	0	0	
Maternal age (years old)	33.0 ± 5.0 (23–42)	33.0 ± 4.0 (23–41)	0.88
Parity	0.50 ± 0.70 (0–3)	0.60 ± 0.80 (0–3)	0.94
Pre-gestational BMI (Kg/m^2^)	21.0 ± 3.4 (15.8–34)	21.0 ± 3.7 (16–32)	0.71
Gestational age at birth (weeks)	39.0 ± 1.1 (37–41)	39.0 ± 1.0 (37–41)	0.13
Birth weight (g)	3148.9 ± 348.0 (2536–3948)	3070.9 ± 316.0 (2592–4440)	0.98
Sex ratio (male/female)	1:1	1:1	0.61
Placenta weight (g)	569.9 ± 103.1 (410–820)	582.0 ± 107 (205–1544)	0.71
Weight ratio of fetus to placenta	0.18 ± 0.030 (0.13–0.26)	0.1 9 ± 0.030 (0.11–0.52)	0.73
Weight gain at 20 weeks of gestation	−0.29 ± 2.8 (−6.9–−4.7)	3.2 ± 2.5 (−4.8–9.0)	0.0001
Net weight gain (Kg)	8.7 ± 3.5 (0–15.6)	10.0 ± 3.3 (3.3–19.4)	0.011
Weight gain from the nadir (Kg)	15.0 ± 4.9 (6–25.6)		

HG, hyperemesis gravidarum; BMI, body mass index; Values are expressed as mean ± standard deviation (SD) (minimum–maximum).

**Table 2:** Clinical background of mothers with HG.

Group	HG (*n* = 34)
Onset of HG (gestational age in weeks)	9.0 ± 2.0 (6–15)
Weight loss (Kg)	6.3 ± 2.8 (0–14)
Weight loss ratio (%)	8.5 ± 3.8 (4.0–18.2)
Duration of admission (days)	27.0 ± 18.0 (8–90)

Values are expressed in mean ± SD (minimum–maximum).

**Table 3:** Values of the fetal growth parameters in the HG group and control group.

Fetal Growth Parameters	Gestational Age (Weeks)	HG (*n* = 34)	Control (*n* = 69)	*P*-Value
	20	0.33 ± 0.8	0.09 ± 0.69	0.12
BPD (SD score)	30	0.26 ± 1.1	0.35 ± 0.82	0.63
	36	0.17 ± 0.89	0.33 ± 0.74	0.35
	20	0.33 ± 1.1	0.47 ± 0.96	0.53
AC (SD score)	30	0.15 ± 1.1	0.32 ± 0.97	0.44
	36	0.22 ± 0.67	0.40 ± 0.86	0.30
	20	−0.24 ± 0.7	−0.02 ± 0.8	0.17
FL (SD score)	30	−0.11 ± 0.84	−0.003 ± 0.86	0.55
	36	−0.2 ± 0.91	−0.045 ± 1.1	0.48
	20	0.17 ± 0.85	0.10 ± 0.69	0.67
EFW (SD score)	30	0.09 ± 0.86	0.05 ± 0.75	0.79
	36	−0.19 ± 0.75	−0.16 ± 0.73	0.81

BPD, biparietal diameter; AC, abdominal circumference; FL, femur length; EFW, estimated fetal bodyweight. Values are expressed in mean ± SD.

**Table 4:** Association between maternal weight gain and fetal growth parameters at prespecified stages.

		HG Group Weight Gain (*n* = 34)				Control Group Weight Gain (*n* = 69)		
GW	Correlation Coefficient = r (*p* Value)	Nadir 20 Weeks	Pre-Gest 20 Weeks	20–30 Weeks	30–36 Weeks	Pre-Gest 20 Weeks	20–30 Weeks	30–36 Weeks
20	BPD	0.47 (0.0048)	0.38 (0.02)			0.037 (0.76)		
	AC	0.18 (0.30)	0.17 (0.42)			0.024 (0.85)		
	FL	0.23 (0.18)	0.26 (0.12)			0.128 (0.88)		
	EFW	0.36 (0.037)	0.03 (0.86)			0.026 (0.83)		
30	BPD			0.04 (0.98)			0.23 (0.06)	
	AC			0.10 (0.58)			0.006 (0.96)	
	FL			0.18 (0.31)			0.21 (0.82)	
	EFW			0.049 (0.79)			0.18 (0.15)	
36	BPD				0.07 (0.70)			0.049 (0.68)
	AC				0.25 (0.17)			0.34 (0.0045)
	FL				0.08 (0.67)			0.13 (0.0045)
	EFW				0.06 (0.75)			0.27 (0.027)

GW, gestational weeks; Pre-gest: Pre-gestational body weight.
